# Railroad Surgeons

**Published:** 1875-04

**Authors:** 


Railroad Surgeons.—The Philadelphia and Erie Division has an arrangement with twenty competent surgeons in various cities along its line, to attend all employees and others injured on or about the line of road. The nearest physician attends for one visit, and then the injured person can select any one of the twenty, and be removed to the town where he lives, and receive his attendance free.—*Medical and Surgical Reporter.*

"That dog of yourn flew at me this morning and bit me on the leg, and now I intend to shoot it the first time I see it." "The dog is not mad." "Mad! I know he is not mad. What's he got to be mad about? It's me that's mad."

